# Principles and application of LIMS in mouse clinics

**DOI:** 10.1007/s00335-015-9586-7

**Published:** 2015-07-25

**Authors:** Holger Maier, Christine Schütt, Ralph Steinkamp, Anja Hurt, Elida Schneltzer, Philipp Gormanns, Christoph Lengger, Mark Griffiths, David Melvin, Neha Agrawal, Rafael Alcantara, Arthur Evans, David Gannon, Simon Holroyd, Christian Kipp, Navis Pretheeba Raj, David Richardson, Sophie LeBlanc, Laurent Vasseur, Hiroshi Masuya, Kimio Kobayashi, Tomohiro Suzuki, Nobuhiko Tanaka, Shigeharu Wakana, Alison Walling, David Clary, Juan Gallegos, Helmut Fuchs, Martin Hrabě de Angelis, Valerie Gailus-Durner

**Affiliations:** German Mouse Clinic, Institute of Experimental Genetics, Helmholtz Zentrum München - German Research Center for Environmental Health (GmbH), Ingolstädter Landstr. 1, 85764 Neuherberg, Germany; Mouse Informatics Group, Wellcome Trust Sanger Institute, Hinxton, Cambridge, Cambridgeshire CB10 1SA UK; Institut Clinique de la Souris - ICS, 1 rue Laurent Fries, BP 10142, 67404 Illkirch Cedex, France; RIKEN BioResource Center, Kouyadai 3-1-1, Ibaraki, 306-0074 Japan; Mary Lyon Centre, Medical Research Council Harwell, Harwell Science and Innovation Campus, Harwell, Oxfordshire OX11 0RD UK; Mouse Biology Program, University of California, Davis, 2795 2nd Street, Suite 400, Davis, CA 95618 USA; Department of Molecular and Human Genetics, Baylor College of Medicine, One Baylor Plaza, Houston, TX 77030 USA; Chair for Experimental Genetics, Life and Food Science Center Weihenstephan, Technische Universität Munich, Freising-Weihenstephan, 85354 Munich, Germany; Member of German Center for Diabetes Research (DZD e.V.), Neuherberg, Germany

## Abstract

Large-scale systemic mouse phenotyping, as performed by mouse clinics for more than a decade, requires thousands of mice from a multitude of different mutant lines to be bred, individually tracked and subjected to phenotyping procedures according to a standardised schedule. All these efforts are typically organised in overlapping projects, running in parallel. In terms of logistics, data capture, data analysis, result visualisation and reporting, new challenges have emerged from such projects. These challenges could hardly be met with traditional methods such as pen & paper colony management, spreadsheet-based data management and manual data analysis. Hence, different Laboratory Information Management Systems (LIMS) have been developed in mouse clinics to facilitate or even enable mouse and data management in the described order of magnitude. This review shows that general principles of LIMS can be empirically deduced from LIMS used by different mouse clinics, although these have evolved differently. Supported by LIMS descriptions and lessons learned from seven mouse clinics, this review also shows that the unique LIMS environment in a particular facility strongly influences strategic LIMS decisions and LIMS development. As a major conclusion, this review states that there is no universal LIMS for the mouse research domain that fits all requirements. Still, empirically deduced general LIMS principles can serve as a master decision support template, which is provided as a hands-on tool for mouse research facilities looking for a LIMS.

**Electronic supplementary material:**

The online version of this article (doi:10.1007/s00335-015-9586-7) contains supplementary material, which is available to authorized users.

## Introduction

Most generally, Laboratory Information Management Systems (LIMS) may be defined as software tools with implemented features that support processes conducted in modern laboratories. Usually, this involves functions like sample tracking, data capture and data management, and some sort of workflow management. Additional specialised functionality like Electronic Laboratory Notebook (ELN), Scientific Data Management System (SDMS) and Enterprise Resource Planning (ERP) tools may be included in LIMS, possibly as optional modules. Many commercial vendors offer LIMS solutions for industry and test laboratories that operate in a highly regulated environment. Typically, these systems are highly customisable and adaptable to user-defined processes and offer standard instrument interfacing protocols, e.g. ASTM E1394 (ASTM International 1997). However, such LIMS are not subject to this review, as it is not meant to be a case study or a questionnaire-based feature comparison of available LIMS. It rather follows an empiric approach by trying to derive general principles from a limited selection of LIMS descriptions, provided by seven large-scale mouse phenotyping facilities (*mouse clinics*).

Such mouse clinics are predominantly running in an academic environment. In this field, mice or mouse-derived samples (blood, urine, tissue) serve as specimens that are subjected to a series of phenotyping procedures. Individual mouse-specific demographic attributes, e.g. sex, genotype, lineage and allelic composition, are required to be linked to captured data throughout the whole process in order to allow subsequent data analysis. Hence, in this field, LIMS have to offer livestock and breeding functionality in addition to standard LIMS features.

In the academic domain, some custom mouse husbandry systems have been developed and published in the past, e.g. LAMS (Frank et al. 1991), MouseNet (Pargent et al. 2000), MICE (Boulukos and Pognonec 2001), MouseBank (Hopley and Zimmer 2001), MUSDB (Masuya et al. 2004), MouseTRACS (Ching et al. 2006), MausDB (Maier et al. 2008), LAMA (Milisavljevic et al. 2010) and JCMS (Donnelly et al. 2010), ranging from pure mouse management systems to integrated mouse LIMS. Certainly, commercial mouse LIMS or colony management products are also available. However, these are not discussed here, as the review does not intend to provide a mere product comparison but rather aims to enable readers to evaluate LIMS solutions by themselves, by providing empirically supported mouse LIMS principles and decision criteria.

## Principles of LIMS

### A generic business process model for mouse clinics

When trying to describe general LIMS principles for mouse clinics, it seems best to first make an inventory of operational activities performed in mouse clinics. Ideally, a LIMS would offer functions to support all those activities.

Thus, we suggest a universal business process model for mouse clinics, where distinct activities can be described on a high level as abstract processes. For instance, “*Mouse Import*” can be viewed as a generic process that involves the physical import of mice from an external source into a mouse clinic including registration of matching mouse entities in the respective LIMS. Independent from many different ways this task may be performed in different mouse clinics and implemented in different LIMS, the process would describe the same activity.

The proposed business process model is composed of the following processes that can clearly be distinguished:

“Request management”: all activities that deal with internal or external phenotyping and/or cryopreservation requests made to a mouse clinic. This may include requester/customer relationship management (CRM activities).

“Project definition”: all activities that are performed to define project information that is necessary to successfully run a project as requested.

“Resource management”: all activities dealing with management of available capacities and resources, including personnel, lab space, cages and instruments (ERP activities).

“Long-term scheduling”: all activities that are performed to overlay required resources and available capacities for existing and future projects. This is done to identify project time slots and to tentatively allocate resources to future projects. Long-term Scheduling can be done using anonymous projected animal numbers. Thus, it can be done long before the actual mice are available. The time range of long-term scheduling is weeks to months ahead of the planned tasks happening.

“Transgenic work”: all activities that involve generating genetically modified mice.

“Mouse production”: all activities that involve production of mice or mouse cohorts for a specific purpose or use in a project.

“Mouse export”: activities required to export live mice, including shipping management.

“Mouse import”: activities required to import live mice from external sources, including shipping management.

“Strain archiving”: activities related to cryo-archiving mouse strains.

“Mouse scheduling”: activities that deal with the allocation of particular mice to a specific purpose or to use in specific procedures. It may also involve the assignment to experimental subgroups. In contrast to long-term Scheduling, this process requires real mice, not just anonymous mouse numbers. The time range of this kind of scheduling is days to weeks.

“Resource allocation”: activities that finally allocate resources to projects in the near future (typically current or next week). This process determines who is intended to perform a procedure on which particular mice on which day.

“Phenotyping”: activities that involve the actual mouse phenotyping, including data capture.

“Data validation”: activities that deal with data validation and quality control (QC). These activities can typically occur during several steps of data capture and data processing.

“Data analysis”: activities performed to analyse acquired data in order to obtain a usable result. Statistical methods and data visualisation are typically applied in this process.

“Result annotation”: activities that involve interpretation of data and results as well as the storage of result annotations as a basis for result reporting.

“Result reporting”: activities that deal with reporting of project results and interpretation using different media (print, web, presentations, publications).

“Data export”: all activities that involve export of raw or derived data.

“Mouse management”: an overarching process that deals with all activities to keep and maintain mouse colonies.

“Sample management”: an overarching process that deals with obtaining, tracking, identification and management of samples and sample attached metadata.

“Genotyping”: an overarching process that deals with determining mouse genotype information from mouse samples.

“Sample archiving”: activities that deal with reliable storage, tracking and retrieval of samples in a cryopreservation archive.

“Project reporting”: an overarching process that involves creating reports on projects for funding agencies and administration. It may also include business intelligence (BI) activities.

“Project controlling”: an overarching process that deals with tracking the status of single or multiple projects throughout project lifetime in order to identify project blockers or necessary action.

“Health monitoring”: an overarching process that deals with monitoring and documentation of animal health in order to maintain a certain sanitary status of a facility, to ensure animal welfare and to enable fast response to animal welfare issues.

“Cost accounting”: an overarching process that deals with attributing costs to particular tasks.

“Invoicing”: a process that deals with sending out project-based invoices and tracking their completion status.

**Fig. 1:**
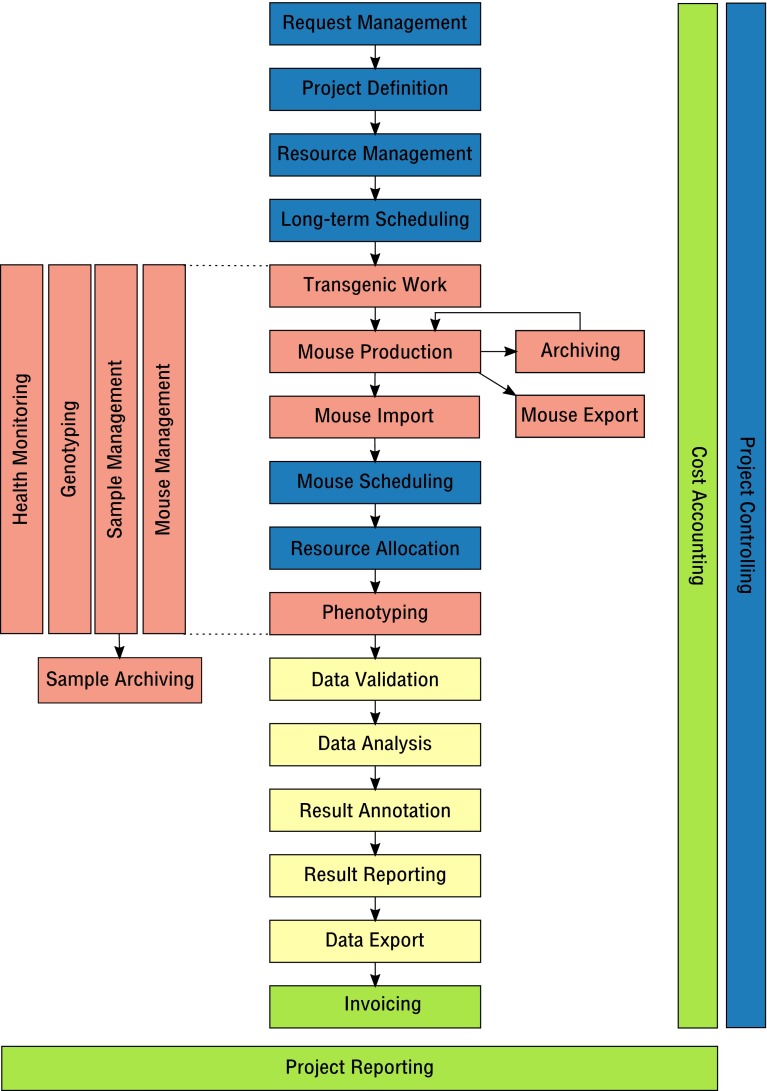
A business process model for mouse clinics. Shown as *coloured boxes* are operational processes that are performed in mouse clinics (described in text). A particular project is run through the processes from *top* to *bottom*, as indicated by *arrows*. Archiving may provide a loop, where a project can be continued later or an independent, derived project can start. Arrow-connected processes may be performed optionally in a mouse clinic or a mouse facility, allowing the application of the model to virtually any facility. Lateral processes accompany a particular project throughout sequential, arrow-connected processes, as indicated by the larger *horizontal* and *vertical* boxes. Different *colours* represent different process families (*blue* process management, *red* working with mice & samples, *yellow* data analysis, *green* finance & reporting)

Having defined the unique processes, we suggest a business process model for mouse clinics (Fig. 1) that describes how all these processes are aligned and how they interface with each other in order to represent the operational activities performed in a functional mouse clinic. In the suggested process model, some processes can be considered as optional, allowing the procedural description of any mouse clinic—even if not all activities are actually performed. For instance, not every mouse clinic might need a process for handling external requests. On the other hand, the process model could even be applied to a mere mouse breeding facility, where mice are just bred and delivered for external use. Accordingly, a LIMS consisting solely of an animal management module would suffice to support the operational activities of such a facility.

### Process-based mouse clinic operations allow modular and pragmatic LIMS solutions

The processes defined above—if effectively installed—are suited to run any mouse facility, no matter how these processes are actually implemented in detail. Hypothetically, a mouse clinic could even be run without a LIMS by implementing every process with simple surrogate technologies like whiteboards, spreadsheets or E-Mail.

Since the processes are independent, either of those can be implemented as a LIMS module or in an alternative way. As a consequence, this allows pragmatic solutions using LIMS that do not cover all operational activities. In fact, LIMS found in mouse clinics are typically composed of a mixture of “real” LIMS modules and complementary non-integrated technologies, mostly spreadsheet files, shared file systems and E-Mail to support processes not covered by the LIMS.

### A classification of LIMS principles

**Fig. 2:**
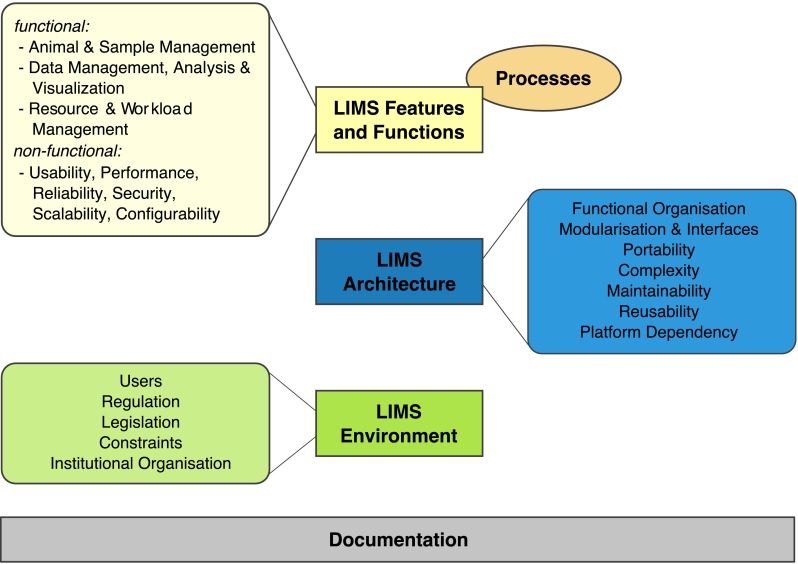
A classification of LIMS principles. For better overview, the figure illustrates the three major areas of LIMS principles (*small boxes*) and the respective properties that can be assigned to these areas (*larger boxes*). LIMS features and functions should support actual processes

Taken into account LIMS descriptions from different mouse facilities all over the world, including those described in this article, three major areas could be identified into which LIMS principles can be classified: LIMS features and functions, LIMS architecture and LIMS environment (Fig. 2).

Although partially overlapping, these areas represent different unique perspectives or stakeholders in a LIMS decision process. Therefore, in any LIMS decision process, a careful stakeholder analysis should be performed. Usually, stakeholders not only involve scientists, technicians and animal care takers but also project managers, veterinarians and people from IT and financial departments. In general, all stakeholders should be involved in a LIMS decision process at an early stage.

#### Features and functions

LIMS features and functions in most cases can directly be correlated with particular processes. However, unless a LIMS is strictly process driven, there is no 1:1 mapping. For instance, basic animal management functions are required in different processes, e.g. *Transgenic Work*, *Mouse Production*, *Mouse Import* and *Mouse Export*. In the following classification of LIMS functions, associated processes are listed.

LIMS functions are certainly most important from a user’s point of view, since users work with the system on a regular basis and the system has to support their daily work. Typically, users are scientists, technicians, animal caretakers and project supervisors. Here, we describe a comprehensive set of features defining the functional requirements of a LIMS. For overview, we summarise functions on a higher level here. Functions are listed in more detail in the supplemental LIMS decision support catalogue.

##### Basic animal- and sample management-related features and functions

Any mouse-enabled LIMS will have to implement features that support daily work with mice and samples, e.g. animal & sample management, animal tracking, breeding & genealogy support and printing functions. Functions in this category are associated with these processes: *Transgenic Work*, *Mouse Production*, *Mouse Import*, *Mouse Export*, *Sample Management*, *Sample Archiving* and *Genotyping.*

##### Scientific data-related features and functions

Functions in this category are used to handle data, e.g. data capture and storage, data analysis, statistics & data visualisation, data QC functions, data annotation, data reporting, data export and interfaces to public databases. They are associated with these processes: *Phenotyping*, *Data Validation*, *Data Analysis*, *Result Annotation*, *Result Reporting* and *Data Export*.

##### Workflow-related features and functions

Functions in this category are used to manage projects, e.g. request management, project management, scheduling functions, resource management (ERP functions), project controlling and workflow customisation. They are associated with these processes: *Request Management*, *Project Definition*, *Resource Management*, *Long*-*term Scheduling*, *Mouse Scheduling*, *Resource Allocation* and *Project Controlling*.

##### Non-science/administration-related features and functions

Functions in this category are used to cope with non-science and administrational issues, e.g. cost/financial controlling & reporting, invoicing, project reporting, business intelligence, animal licence controlling & reporting to authorities, animal welfare documentation and multi-site capabilities. They are associated with these processes: *Cost Accounting*, *Invoicing*, *Health Monitoringand Project Reporting*.

##### Non-functional features

In contrast to functional features, describing *what* the LIMS should provide in terms of its original operation purpose, non-functional features describe *how* the LIMS should behave in more general, technical terms. Non-functional features are not specific to a certain LIMS or domain but could rather be applied to any system. LIMS aspects like security, response time, speed, scalability, data integrity, data archiving, audit trail and reliability are examples of non-functional features.

#### Architecture and technical aspects

LIMS architecture can be defined on two different levels. On a functional level, it describes how LIMS functional modules are organised, how they interact with each other and how they cover the operational processes of a given facility. Involved stakeholders would be on a management level.

In contrast, technical LIMS architecture defines—as the term implies—on which technologies the system is built and how these technologies interact with each other to form a whole LIMS. Typically, stakeholders are IT, management and administration.

##### LIMS workflow coverage

Workflow coverage defines to which degree all processes are covered in an integrated LIMS. We showed above that LIMS covering only distinct processes are possible. Mostly, LIMS workflow coverage is a matter of available resources and priority and thus a management decision.

##### Technical integration

LIMS technical integration level is defined by the homogeneity of used technologies and interfaces. This is strongly influenced by the development history of a LIMS, which in turn may be influenced by stability of funding and resources, including staff size. Funding instability may lead to less integrated LIMS with more or less independent legacy or third-party modules. While this may not necessarily be a bad solution in terms of functionality, it probably is not good in terms of maintenance, as mix of different technologies has to be matched by the expertise portfolio of a team.

##### LIMS architecture

In terms of the above-discussed processes, a modular LIMS would provide the best overall architecture, since functionality would be implemented independent from each other in different modules, using well-defined inter-module interfaces. As every module could be adapted to changing purposes independently, this architecture is very flexible. In contrast, a monolithic LIMS architecture usually allows less flexibility, as there may be many complex ties in the code.

##### Platform

Web-enabled LIMS are probably the solution of choice compared to classical Desktop applications, since they provide platform-independence on the client side. Using modern technologies, in particular AJAX, the user experience of web-enabled LIMS can be comparable to Desktop applications. In academic in-house LIMS development, the programming language in many cases is strongly influenced by available team expertise. However, it is a strategic decision that should be critically reviewed in terms of sustainability. This is also true for the choice of the database management system (DBMS), in case no central database operation group or service is available on site.

##### User experience

User experience is a major factor that should be considered early in a LIMS decision process. In a strictly workflow-driven LIMS, users always have to follow a step-by-step procedure determined by a rigid process. In contrast, a free navigation user concept allows more flexibility; however, it requires a higher training level to ensure quality. The user interface ideally would be self-explanatory or offer context-specific help.

Access to particular functionality is most often attached to user roles, e.g. scientist, animal care taker, manager etc.

#### LIMS environment: institutional policies and other major settings

Using the term “LIMS environment”, we subsume all factors that influence the context and circumstances in which a LIMS operates. These are mostly set by high-level corporate and management decisions and strategies. However, also users can become stakeholders here, depending on how much they are able to influence LIMS environment.

At first, two central paradigms can be observed in different institutions: “*workflow follows LIMS*” versus “*LIMS follows workflow*”, where “workflow” subsumes the overall way work is done. To follow the “*workflow follows LIMS*”, paradigm means that the LIMS prescribes the way work is done. As a consequence, it can also limit the operational activities of a facility, in case it does not functionally support requirements. “*LIMS follows workflow*” allows means that operational requirements come first and the LIMS has to be adapted. Following this paradigm allows more flexibility, however, it requires much more resources for custom development. We will see examples for both paradigms in the “LIMS examples” section.

Related to this paradigm is the primary decision whether to buy a commercial LIMS, to outsource custom LIMS development or to establish in-house development capacities. Naturally, not only flexibility and independence but also costs are correlated with this decision.

In a particular institution, availability and sustainability of resources will strongly influence LIMS environment. A fancy and expensive LIMS that requires a lot of maintenance resources may work worse than a lean LIMS solution in an environment with restricted resources.

Another LIMS environment factor is the level of regulation. Next to administrational or local governmental regulations, cooperation partners or customers may set requirements for the LIMS, e.g. compliance with FDA Title 21 CFR Part 11 (the United States Food and Drug Administration (FDA) regulations on electronic records and electronic signatures).

LIMS users are part of the LIMS environment in different ways. The level of user fluctuation, user heterogeneity, common language, staff training level, quality awareness and user compliance will influence the need for data curation and support.

The organisation of an institution also is part of LIMS environment. In terms of LIMS requirements, there is much difference between a centralised and strictly organised facility—using well-defined processes and SOPs—and a decentralised organisation with more or less independent user groups.

## LIMS examples and lessons learned from mouse clinics

The principles of mouse LIMS described in this review are claimed to be universal and not specific to a particular LIMS. However, we believe that the LIMS environment as described above strongly influences the implementation of a LIMS in a facility. In the following sections, we provide “real-world” LIMS examples, contributed by seven mouse clinics, which all are members of the International Mouse Phenotype Consortium (IMPC, http://www.mousephenotype.org) (Brown and Moore 2012). They all contain short LIMS descriptions and highlighted features considered to be of major importance in their respective environment. Moreover, they describe important lessons learned during the implementation and use of their LIMS. The LIMS examples provided here are original, non-edited contributions, which are not intended to be used for feature comparison, but rather to serve as an empiric source for LIMS principles.

### Example 1: Mouse Informatics Group, Wellcome Trust Sanger Institute (WTSI), UK

The WTSI Mouse Database has been in existence for about 8 years and covers all aspects of high-throughput mouse production including mouse husbandry, freezing and thawing of cryogenic resources, phenotyping (data and images), data visualisation, reporting and export of resources (both mice and phenotypic data) all under strict UK Home Office licensing. At any given point, it is possible to generate full cage accounting on a per-month basis. This will show how many cages have been used per month attributed to which financial cost code. Users are also able to see how many cages have been used per-week/month per line. The LIMS is built and maintained by a core team of 7 developers using Java and the Spring Framework and has an active user base over 200 scientists, technicians and managers across the institute. The underlying database is currently holding just over 50,000 live mice and over its lifetime has tracked more than 2 million.

Our system is available as a fully hosted, web-delivered service (like Gmail). An organisation can, for a yearly fee, have full access to a LIMS system based on the one developed at WTSI. This allows customer organisations to make use of the features of the WTSI software but without having to purchase servers and database licences of their own, or have dedicated support staff to maintain and backup their data. Our LIMS solution is hosted in two geographically diverse data centres to provide high availability and resilience to failure. The LIMS application has been security audited, and access to customer’s data is protected by a number of tried and tested mechanisms. This approach allows us to offer a very competitive price when compared to the total cost of ownership of alternative LIMS systems.

One major feature of our LIMS is the ability to create bespoke user-defined data entry forms. These enable a template to be created exactly as a user defines with all the field types they need to collect, e.g. data type, default values and numerical ranges. The template has real-time preview, so the finished form that will be filled in can be evaluated at the point of creation. This enables anyone to create or modify the data required for collection as and when that necessity changes, without needing IT support or bespoke features adding to the main application. Once these forms are created they can be used stand-alone to capture the data from a particular phenotypic assay, e.g. X-ray, or can be embedded within other LIMS pages where they can support the collection of required metadata, e.g. CRISPR/Cas Concentration. By expanding the capability outside of just collecting phenotypic data, we can empower the LIMS users to create and modify their own data collection requirements as the scientific technology and techniques advance over time.

A further extension to the collection of the data is that these forms can be reported out by creating “Oracle views”. Views are simply the representation of SQL statements that are stored in memory, so that they can be easily re-used. These are created in the database by joining a mouse level report, which contains all the standardised mouse information (Gender, Genotype, Genetic Background etc.), and the particular columns recorded in a form. Each assay can be reported on by the user, and all columns in the form can be used as filters. The resulting data can be rendered as a stand-alone searchable table on a reporting page or embedded on another page that requires real-time data, e.g. List of mating’s within a mouse holding room. This tabular report can also exported out as csv format for offline use. The phenotypic data is also visualised by means of an internal heatmap where by each line and assay is its own distinct cell within a grid. Each assay is split by each protocol (variation in assay, e.g. anaesthetic or diet) to show individual parameters and each parameter can be rendered as a graph. The user will evaluate the significance of the data based on the reference range and local controls to decide whether the data are deemed significant. MP terms and a comment can also be assigned to each graph. Any call made by the user is usually supported by an initial automated call that is generated overnight and will flag each graph’s significance by a coloured corner (red-significant, blue-not significant).

#### Lessons learned

Responding quickly to changes in scientific working practices and new technologies puts an additional strain on resources required to maintain a high-throughput application. We have a brokering system in place that allows the senior managers to give the team a roadmap of upcoming development requirements for new functionality or changes to existing modules organised into a prioritised list. Into this, we will add in our own priorities for the software (e.g. module refactors, framework updates, major bugs, updates to team standards) to create the roadmap for the year ahead. As the brokering meetings typically occur every 4 months, this allows that roadmap to be flexible enough to respond to changes without having the team constantly switching from one piece of development to another in quick succession. This also enables us to plan a couple of modules of work ahead of time, meaning that the up-front business analysis can be done before development begins. The benefit of this is that in most cases, the specification of what is required for the user has been thought about for a good length of time and is relatively clear. The actual development is agile in nature so that as the work progresses the key user/stakeholder (who we meet a couple of times a week) can respond to issues or functionality and the resulting changes can be implemented quickly with very little impact.

### Example 2: Japan Mouse Clinic (JMC), RIKEN Bioresource Center, Japan

Japan Mouse Clinic (JMC) has been set up in 2008 (Wakana et al. 2009) by the expansion of the mouse phenotyping platform of the large-scale ENU Mutagenesis Program in RIKEN in which operations had been managed by a LIMS termed as Mutagenesis Universal Support DataBase (MUSDB) (Masuya et al. 2004). In JMC, data operations in the data capturing (mouse husbandry, colony management, cryopreservation of sperms and eggs, data capturing from the phenotyping platform, genotyping of polymorphic markers and linkage analysis of phenotype and genetic makers) are supported by MUSDB. The statistical phenotype data analysis pipeline is provided by the independent software application, termed as “Pheno-Pub” (Suzuki et al. 2013) which supports a series of data-handling and Web-publication tasks in the large-scale phenotyping. In addition, Web-publications of the experimental SOPs are supported by a protocol database, termed as “SDOP-DB” (Tanaka et al. 2010). From 2011, JMC participated in the IMPC as one of the primary phenotyping pipelines for IKMC mutant lines.

In 2013, JMC decided to replace its LIMS from MUSDB to the modified version of WTSI Mouse Database (described above). The original software codes of WTSI Mouse Database were transferred from WTSI. Then, the modification and operation of the database is performed in the local network of the RIKEN BioResource Center. Currently, the modified version of the software is termed as “RIKEN LIMS”. The transfer of the LIMS operation has started late 2014. We gradually replace MUSDB operations to RIKEN LIMS with turning over of animals in the JMC’s animal facility. RIKEN and WTSI are now planning to share the modified software codes.

#### Lessons learned

For the long-term operation, there appear serious problems on the sustainability of MUSDB: (1) client applications are developed to work only on the previous versions of Windows^TM^ OS platform and (2) the database table structure, which has fixed columns for specific measurement parameters, cannot cope with changes of experimental SOPs, which are needed for continuous operation of JMC. Therefore, replacement of LIMS was one of the indispensable plans in JMC.

Original features of WTSI Mouse Database, which allows customisation of a lot of data entry fields for centre-specific attributes (e.g. rooms of animal facility, phenotypic SOPs, options for measurement parameters and so on), help the transitions of LIMS operations from MUSDB to RIKEN LIMS in JMC. However, it turned out that several “customs” in the core procedures in the mouse husbandry were unchangeable. For example, in the JMC, both of operations of animal caretakers and phenotypers are deeply dependent on the information, which is represented in names of animals (i.e. sex, generation in the colony and sequential number in a generation). We found that changes of the naming system of animals were inferred to affect seriously to the efficiency of the total operations in the JMC. Therefore, we modified some parts of functions for basic husbandry operations in the software. In addition, we added some “useful” functions for phenotyping (e.g. quick starting of phenotyping data capture and universal data browser).

### Example 3: Baylor College of Medicine (BCM), Houston, TX, USA

In 2011, Baylor College of Medicine adopted the WTSI Mouse Database application (as described above) for tracking of production and phenotyping data in the Knockout Mouse Phenotyping Program (KOMP2) project. With over 5 years of development, the application had been thoroughly tested and was evident as no major technical issues were found to be obstacles during this transition period.

In the initial stages of migration the application was well received by the initial wave of users. However, it was noted quite early by management that a large amount of time would have to be invested for full adoption of the application for a project as large as KOMP2.

Most notably the scheduling and collection of data by phenotypers and machine outputs generated across several phenotyping cores at BCM. The case for non-machine output was easily handled by the application as it allowed for the easy creation of custom parameters and data capture forms (DCFs). These procedure DCFs would eventually be created to mirror the protocols and specifications set by the IMPC such as metadata parameters with pre-defined dropdown options.

Machine output generated by phenotypers arrived in multiple formats and file extensions such as CSV, PDF and Images. For the exception of a few procedures, most machine outputs required a learning curve by IT to be able to format or compute the correct format expected by the IMPC. Fortunately, the learning curve was minimal with regards to importing data to the application as the WTSI application can be configured with minimal effort to accept data in batches using the mouse barcode and date of procedure.

However, although the application excelled in many features, its overall robustness and scale would be viewed as intimidating to a small subset of the users. This would mainly be overcome through time, large effort in documentation, and various training sessions. In doing so meant adopting the “workflow follows LIMS” paradigm as described in this publication at our institute for the early stages of the project.

BCM began transitioning into “LIMS follows workflow” as the project progressed and full understanding of the WTSI application was learned at BCM. Recent development by the BCM informatics group has evolved a modified version of the WTSI application that will be termed for the purpose of this publication as “BCM LIMS”. This modified version was created to take into account differences in workflow between centres, to incorporate decisions and strategies set by stakeholders and to facilitate the reporting and tracking of operational activities.

#### Lessons learned

A large investment of time will be required to introduce IT members to a project of this scale. Without the understanding of the business logic of the project, the individual will find it difficult communicating between biologists and deliver on biologist requests.

At the initial point of data collection IT encountered inconsistent records. These records were captured in excel sheets and machine outputs that varied in format. An investment of time to learn biologist tools and machines was required to evaluate data collections, machine data extraction, formats, and importation of data to central database. In general, machine output did not come with documentation, so communication between biologist and IT was vital to meet objectives. In addition, decisions made would have to be implemented and followed by training of biologist staff.

Securing data integrity can be accomplished by defining data collection protocols and QC checkpoints at the initial stages of a project. For example, BCM introduced QC boundaries that would be used prior to data submission to reduce QC flags raised by the IMPC. These boundaries were set using publications and control data generated by the project to distinguish impossible values from abnormal phenotypes. The use of data visualisation tools greatly benefitted management and biologist in the tracking of data.

A resistance to change will always be present. We found that gradual steps, documentation, creation of videos and large effort in training were essential to break conventional collection methods to transition to LIMS application.

### Example 4: Institut Clinique de la Souris (ICS), France

The ICS LIMS for the phenotyping platforms (“BIOX”) is a custom-developed web-based application. It is based on a modular design to capture administrative and scientific data and is fully compliant with the IMPC export schemes. It is working in interaction with other parts of the ICS information system through web services.

#### Key features

*Project management*: Biox enables semi-automatic project tracking with workflow description and scheduling information. Projects managers and administrative staff rely on it for planning and reporting.

*Animal facility management*: A dedicated application is used to manage lines, mice and genomic data. It integrates well-defined roles and permissions for the interactions between scientists and technicians. Data interactions with Biox are performed through web services.

*Experiment and data capture*: Biox can capture data and experimental conditions for more than 60 standardised experiments. Different data capture profiles allow adapting configuration on projects and client needs. Data can be loaded into LIMS using Excel templates, proprietary data file decoders or via direct connection from equipment.

*Quality control*: Biox includes format validation and range checking regarding reference ranges calculated for the corresponding workflow. Outlier data points out of expected biological ranges are flagged. After double-check, data are validated by the service responsible and can’t be modified anymore. Finally, missing mandatory values are also checked during loading and export processes.

*Data analysis, visualisation and extraction*: Standardised statistical analyses are systematically done on each set of data:

- Mean, standard deviation, standard error of mean.
- Comparison between groups is done using Student test or Chi-square test of independence.
- Power of statistical test used and effect size.
- Reference ranges of the corresponding wild type mice are also displayed and graphed.

Automated process exists to automatically generate Mammalian Phenotype Ontology annotations, as well as graphs generation (histograms, scatterplots, time series, boxplots).

*Data export and other systems connections:* Data can be exported not only in standard excel spreadsheets but also in CSV or XML formats for external databases. Biox is connected to several other internal systems through web services: animal facility system (mice information), LDAP (client accounts) and genetic engineering system (mutation information). It also works in interaction with external databases: MGI (gene data), IMITS and IMPC database (data exports).

#### Lessons learned

The ICS LIMS has evolved towards a modular design, which is essential to separate concerns and improve the pace of evolutions of the different parts. The standardised configuration of new tests also avoids custom developments and shortens the time to put them in production.

QC and security are also vital for the reliability of the scientific results and thus for the Institute’s image. They have been taken into account from the start and continuously improved through the years.

### Example 5: Mouse Biology Program, University of California, Davis, USA

The Mouse Biology Program LIMS (MBP-LIMS) was designed and implemented in 2011. The requirements for the MBP-LIMS dictated a custom-built UAMP (Ubuntu, Apache, MySQL, PHP) architecture-based application that followed the workflow, allowed for flexible project and resource management for internal and external projects and interconnected with multiple other legacy systems existing in place such as those for colony management and mutant mouse production. The requirements also stressed ease of interaction for data collection and simple resource re-allocation (e.g. after initiation of a project).

To allow for modular construction and maintenance, the application was built using the CakePHP MVC (Model View Controller) framework. The use of this framework permitted rapid development and implementation of core functionality with a 2-person team over 3 months. One of the major benefits of using this framework has been the built in database access, caching, validation, authentication and access level control (ACL). Particularly useful has been the application of ACL to blinding technicians to mouse genotype and gender information as well as ease of creating functional roles such as technician, supervisor, administrator, investigator, observer and IT. The basic data model was built around projects that were organised by procedures made up of resources (e.g. mice, equipment, technicians, rooms). To address the ease of UI (User Interface) requirements, both project management and data collection revolve around a Google style calendar with all projects and procedures listed for that day/week/month. Procedures can be dragged and dropped to reschedule when resource constraints or conflicts occur, or clicked through to collect data. This application is freely available to the public as an unsupported Git repository under the terms of the GNU General Public License.

#### Lessons learned

##### User interface usability can have a huge contribution to data quality

Collecting high-throughput data can be a mind numbing experience at the technician level and can easily result in poor-quality data. Project management, staging, and organisation all compete for technician attention and distracts them from what should be their focus, collecting a clean dataset. Tools that streamline and assist in the organisation and collection of data are not only good for technician buy-in of an application and are also critical for ensuring good data quality.

Systems that do not follow the physical collection process or are difficult to interact with risk technician opt-out. In this situation, the technician will collect data offline or will avoid using the system altogether. At best, there is the risk of transcription errors while inputting data back into the system. In the worst case, this can lead to technician frustration and antipathy, lost productivity and poor-quality data. To correctly design and implement, a system with a high level of usability requires a close interaction with lab personnel during development, testing and implementation and a willingness on the programmer’s part to understand the process through the technician’s eyes. Any part of the process that causes frustration, lost efficiency or confusion at the technician level is a likely candidate for client side tools or reports.

##### QC tools are most effective at the time of data collection

Most data collected at the bench can only be effectively monitored for quality control (QC) at the time and point of collection. An incorrect or spurious data point can be removed or flagged after collection but the ability to successfully correct it is a very narrow window during data capture. Post-collection QC is still essential, but corrective action is limited to changing the process for future data collection or determining equipment or process failures. Features should exist within the application to catch potentially bad data at the time of capture and flag it for the technician’s attention immediately, giving them the opportunity to correct issues on the spot. This process ensures that the collected data set will be accurate and reproducible.

#### What we would do differently if starting again

##### Manage user expectations better regarding critical functions that can impede workflow

One of our critical requirements for the application was blinding the technicians to sex and genotype information. This caused some initial issues for assays where the technicians felt they needed to know this information in order to perform the assay. We eventually resolved this by un-blinding the vivarium and supervisory staff, so they could manage the workflow while still keeping the technicians collecting the data blinded. In retrospect, it would have been valuable to have the supervisors step through the initial process and recognise that blinding would become an issue. At that point, we could have adjusted our process to compensate and discussed the importance of blinding with the technicians before the application was put in production.

##### Give data analysis a higher priority when developing the application

Having limited resources available to develop the MBP-LIMS, we chose to focus on data collection and workflow management initially rather than data analysis. While data collection for a new assay is critical and needs to be put in place first, data analysis should follow closely in order to detect startup problems with assay processes and procedures. This lag in data analysis led to several procedural issues not being addressed as quickly as they should have been and have affected the early data quality of some assays.

### Example 6: Informatics Group, Mary Lyon Centre (MLC), MRC Harwell, UK

The MRC Harwell LIMS (AnonyMus) has been developed in order to support the work being undertaken within the Mary Lyon Centre. It has been in production use since 2004, undergoing iterative development since its release.

Functionality currently includes support and tracking of all husbandry processes; phenotyping and imaging across a broad spectrum of assays; drop-box data file loading; data reporting, analysis and visualisation tools for real-time QC; mouse production, micro-injection, import, export, archiving and re-derivation; genotyping with automated robotic control and cassette based calling logic; necropsy, histology and histopathology; service request management and MTA’s; competency training; welfare assessments; cage management; UK Home Office licensing and automated scheduling and emailing of data reports and data exports.

The MLC’s operations adhere to a quality management system, which has been ISO 9001:2008 accredited since July 2010.

AnonyMus is a Java web application interfacing with a relational database back end, being developed under Agile software development methods and principles.

There are currently 2.5 million mouse records on the system, with over 100 million data points held within the database.

In recognising that certain areas share common principles and elements, generic designs have been introduced in order improve our implementation efficiencies through reuse.

Configurable elements of user interfaces are stored at the database level, with an underpinning software architecture that dynamically builds rich, user friendly interfaces at run time. This enables new data entry forms or new interactive query reports to be instantaneously provided to users, without the need for any changes to the application code itself.

Phenotyping assay data entry forms can be configured by specifying the underlying data fields and data types, along with what user interface widgets to build at run time. Data export mechanisms automatically detect any data changes that need reporting, sending them to the Data Coordination Center (DCC) as scheduled via XML. Common re-useable activities can be linked into a series of configurable processes in order to build a workflow. This has been used for tracking a wide range of sample management processes such as ear clip biopsy plates, terminal bleeds and tissue and organ collection. A generic and configurable request management console has also been provided which is being used to track both internal and external service requests alongside all associated data capture. This is in use for both biorepository services and histology services, enabling both project and KPI level reporting.

The work being undertaken within the MLC is continually evolving. Breeding strategies change; genotyping methodologies evolve as new assays become available and new and challenging scientific research projects are initiated which require bespoke informatics support.

As such, the LIMS must be reactive and flexible to the unit’s needs and as yet, no “off the shelf” system has been identified which would be sufficiently configurable across all our domains of work. A core team of 5 FTE’s currently support the system itself and the new software implementations required to underpin the unit’s objectives and deliverables.

The AnonyMus system can be made available upon request subject to terms and confidentiality agreement. Support or assistance for its implementation would necessitate a fee.

#### Lessons learned

- The Agile development methodology should have been adopted sooner.
- Fortnightly review meetings with a knowledgeable user group, empowered to make decisions on prioritisation and requirements, is essential.
- A complex system such as ours with cross module dependencies involves a steep learning curve; modularisation with a unified architecture is the ideal.
- Have a mechanism for prioritising rare use-cases alongside new user requirements if back-end support proves complex, high risk or repetitive.
- Re-factoring should occur at an optimal level for critical areas which are at risk.
- It is essential to have highly experienced software engineers embedded within the team, who are invested in the peer review and mentoring processes.
- Porting a LIMS for use elsewhere is a significant undertaking. It must be sufficiently documented and adequate resources and expertise must be made available in order to configure the system and modify code where necessary.

#### What we would do differently if starting again

- Automated testing would be incorporated into all modules, not just those that are new.
- Standards and best practices would be peer reviewed for all implementations.
- The system would be modularised with clear interfaces; minimising complexity and reducing maintenance/modification burden in the long term.
- Provision of better interfaces for configuring generic elements.

### Example 7: German Mouse Clinic (GMC), Helmholtz Zentrum München, Germany

At the German Mouse Clinic, large cohorts of mutant and control mice are either imported from collaboration partners or produced in-house to be systemically phenotyped. This requires sophisticated informatics systems to support coordination of the involved complex logistics as well as to allow storage and analysis of the huge amount of data generated. MausDB, the web-based LIMS of the German Mouse Clinic, has originally been developed in 2006 (Maier et al. 2008) and has been in use in our facility since then. Until 2011, this “basic” MausDB was a pure LAMP (Linux, Apache, MySQL, Perl) system. It provides functions for mouse husbandry and tracking, phenotyping workflow scheduling, phenotyping data capture and storage as well as subsequent automated data analysis using customised scripts written in R (R Core Team 2013) for statistics and visualisation.

The German Mouse Clinic has implemented a Quality Management System for its systemic phenotyping activities, which has been ISO 9001:2008 certified in 2014. In preparation for this, a comprehensive business process model has been developed, covering all processes starting with external request management, mouse import and colony breeding, phenotyping workflow scheduling, data capture, data analysis and visualisation, to results reporting. At the GMC, such a well-defined business process model is essential for planning, coordination, controlling and reporting on more than 100 projects per year (high-throughput primary and secondary screening projects as well as faculty research projects) running in a multi-parallel fashion.

In order to implement full LIMS support for this business process model, the GMC started a large MausDB improvement and integration project in 2011. Since then, MausDB has consequently been re-engineered and supplemented by new software modules, each implementing support for a distinct process. These rather independent software modules have been developed in Java using the Spring and JSF frameworks and provide particular process functionality and interfaces to other modules.

#### Key features

Resource management and controlling is a very important issue at the GMC in order to optimise use of existing capacities. Therefore, a key feature of MausDB is to enable permanent target-performance comparison throughout the whole project. In our LIMS, any kind of standard or custom project can be defined by assigning more than 100 project attributes in the project editor module. As soon as the project starts, progress can be tracked in the project-tracking module, where the comparison between pre-defined tasks and the current status is visualised. This allows easy detection of required next step actions and project blockers. Another example is the project scheduler module, where predicted phenotyping capacities for all tests are compared to actual assigned tests for every week. A simple colour visualisation enables project managers to detect overbooked (yellow, red) or idle capacities (white) at a glance.

A key aspect of MausDB’s overall architecture is full integration of all functional modules in one common LIMS session. In this architecture, the legacy Perl-based “basic” MausDB described above is considered a module just as the new Java-based modules. Therefore, it can be transparently integrated in terms of session management and interfaces, although two different frontends are still in use. This concept allows running this module stand alone, providing a fully featured mouse colony management system for facilities that only need basic mouse husbandry and tracking functions. As a matter of fact, MausDB is the central LIMS of the Helmholtz Center Munich, with 16 independent MausDB installations managing animal colonies for different Helmholtz institutes on campus. Different MausDBs host mice, rats, hamsters and other mammal species. Another 17 MausDB installations are currently running in different mouse facilities worldwide.

The GMC emphasises independence from commercial solutions and the freedom to customise and adapt our LIMS to upcoming requirements at any time. Not least to this fact, MausDB is consequently built on non-commercial components. For instance, the reporting engine combines the free software packages R (R Core Team 2013) and LaTeX (http://latex-project.org/). At the push of a button, it is able to produce comprehensive printed PDF project reports for our customers with 100–200 pages, including statistics and embedded graphs.

So far, MausDB is holding 330,000 mice and their associated demographic and phenotyping data, with an average of about 10,000 live mice at any time. About 110 scientists, technicians, animal caretakers and project managers in the GMC are currently using MausDB.

MausDB is compliant with current EU regulations concerning reporting of animals used for scientific purposes (THE EUROPEAN COMMISSION 2012). MausDB collects all data needed for generation of the required reports at different time points (import, weaning, experimental assignment, culling). A separate reporting module collects and processes all relevant data to generate the report in the format specified by the EU directive. This module is even capable of collecting data from different, independent MausDB installations. At our institution, this allows running separate MausDB installations for different institutes, working groups or animal species without mutual access but still being able to report on the overall animal use.

Currently, the basic MausDB husbandry module is freely available under the terms of the GNU General Public License, as published earlier (Maier et al. 2008); however, support cannot be provided.

#### Lessons learned

##### Generic projects are more suitable to organise work and data than a biological concept like mouse line

In the first years of GMC operation, it seemed natural to organise work and data by mouse lines and respective genes. When projects involved the same gene a second time, we needed to discriminate datasets, which turned out to be difficult with this concept. Therefore, we introduced generic projects as major concept, within which mouse line and gene are just attributes amongst others.

##### Operational flexibility comes by well-defined processes and LIMS modules functionally supporting them

As with many large operations, processes in the GMC changed over the years and in turn, our LIMS had to change as well. An important lesson learned at the GMC was that we are far more flexible and adaptable when using a modular LIMS architecture that supports well-defined processes. Thus, necessary modifications can be restricted to one or two modules without affecting the whole system.

##### Complex logistics is a limiting factor and should be supported by LIMS rather than by distributed spreadsheet files

At the GMC, we learned that logistics can be limiting to operational capacity. Prototyping logistics using spreadsheets is fine, but on the long run, critical business information has to be managed in the LIMS. Only there, it is available in real-time and can be linked to other information.

##### LIMS development and data curation work better if they are performed by different teams

At the GMC, we learned that LIMS development and data curation should be performed by different persons. These activities require quite different skills. Also, having to do both requires a constant trade-off between writing best possible code and doing best possible data curation.

#### What we would do differently if starting again

- More developers would work on LIMS development in the early phase to enable large development steps and best practice solutions from the beginning.
- The LIMS would have a modular architecture, driven by well-defined processes, from the beginning.
- Put more effort in analysing and optimising logistics, using well-defined processes and interfaces, and then include everything in the LIMS (rather than keep using spreadsheet files for some purposes).
- Invest even more time in discussing and prioritising stakeholder requirements.

## Conclusions

This review describes a comprehensive set of principles of LIMS in the mouse research domain, based on experiences made with such LIMS in seven large mouse production and phenotyping facilities. All seven mouse clinics are members of the IMPC consortium (Brown and Moore 2012; Koscielny et al. 2014) and are committed to perform high-throughput mouse colony production and phenotyping according to common IMPC standards, which implies the overlap of LIMS requirements.

However, LIMS descriptions and lessons learned from the different mouse clinics clearly show that very individual LIMS solutions have evolved despite such considerable requirement overlap. The suggested business process model for mouse clinics delivers the theoretical foundation to explain the observed LIMS diversity. On a more practical level, it seems that high-level strategic LIMS decisions and the way a LIMS is chosen or developed in a particular institution are strongly influenced by factors, which are not originally defined by scientific requirements. We have subsumed such factors as “LIMS environment”, a specific set of institutional settings, management strategy, work traditions and local governmental and administrational guidelines.

Using the concept of LIMS environment, we can explain why distinct LIMS solutions can be found in institutions that share very similar scientific requirements and processes but still differ in their individual LIMS environment. These LIMS environments are highly individual for an institution, and there seems to be little chance and need to harmonise them. Hypothetically, an exception would be an institution that is build from scratch and fully adopts LIMS requirements as well as LIMS environment from another institution. We provided one example of an on-going LIMS transfer project, where the Japan Mouse clinic has adopted the WTSI LIMS. However, partial incompatibility of LIMS environment—in this case certain traditions in mouse husbandry—leads to custom modifications of the adopted LIMS.

The intention of this review is not to perform a comprehensive comparison of different LIMS. Such an effort would have required access to third party, including commercial LIMS, which was not achievable for the authors. Furthermore, the authors are not aware of, however cannot exclude, the use of commercial LIMS in the domain of large-scale mouse phenotyping.

As an overall conclusion, there seems to be no universal or generic LIMS that will work perfectly for any purpose or any mouse facility. However, this review can provide a comprehensive overview of general LIMS principles that are empirically supported. These principles are summarised in a hands-on decision support catalogue that can be used to compare or evaluate LIMS alternatives.

## Electronic supplementary material

Supplementary material 1 (XLSX 43 kb)

Supplementary material 2 (DOCX 11 kb)
