# Supplementary material for: Principles and application of LIMS in mouse clinics
Source: Mamm Genome. 2015 Jul 25;26(9-10):467–81. doi: 10.1007/s00335-015-9586-7 (PMC4602070; doi:10.1007/s00335-015-9586-7)
Supplement: Supplementary file 2 — Supplementary material 2 (DOCX 11 kb) [file 335_2015_9586_MOESM2_ESM.docx]

**Supplements**

**Supplement 1: A LIMS decision support catalogue template**A structured, hands-on checklist template in Excel format, specifying requirements on LIMS functions, architecture and LIMS environment, according to principles described in this review. The checklist can be used to evaluate LIMS and intentionally is provided as Excel file, to allow user-specific modifications.
